# Propane-1,3-diyl bis­(pyridine-4-carboxyl­ate)

**DOI:** 10.1107/S1600536810011700

**Published:** 2010-04-02

**Authors:** Iván Brito, Javier Vallejos, Michael Bolte, Matías López-Rodríguez, Alejandro Cárdenas

**Affiliations:** a Departamento de Química, Facultad de Ciencias Básicas, Universidad de Antofagasta, Casilla 170, Antofagasta, Chile; b Institut für Anorganische Chemie der Goethe-Universität Frankfurt, Max-von-Laue-Strasse 7, D-60438 Frankfurt am Main, Germany; c Instituto de Bio-Orgánica ’Antonio González’, Universidad de La Laguna, Astrofísico Francisco Sánchez N°2, La Laguna, Tenerife, Spain; d Departamento de Física, Facultad de Ciencias Básicas, Universidad de Antofagasta, Casilla 170, Antofagasta, Chile

## Abstract

The title compound. C_15_H_14_N_2_O_4_, (I), has a gauche–gauche (O/C/C/C—O/C/C/C or GG) conformation and is a positional isomer of propane-1,3-diyl bis­(pyridine-3-carboxyl­ate), (II). The mol­ecule of (I) lies on a twofold rotation axis, which passes through the central C atom of the aliphatic chain, giving one half-mol­ecule per asymmetric unit. There is excellent agreement of the geometric parameters of (I) and (II). The most obvious differences between them are the O/C/C/C—O/C/C/C torsion angles [56.6 (2)° in (I) and 174.0 (3)/70.2 (3)° in (II) for GG and TG conformations, respectively] and the dihedral angle between the planes of the aromatic rings [80.3 (10)° in (I) and 76.5 (3)° in (II)]. The crystal structure is stabilized by weak C—H⋯ N and C—H⋯ O hydrogen bonding.

## Related literature

The title compound can be used as a nucleophilic tecton in self-assembly reactions with metal centres of varying lability. For conformation definitions see: Carlucci *et al.* (2002 ▶). For related structures, see: Brito *et al.* (2010 ▶); Chatterjee *et al.* (2004 ▶).
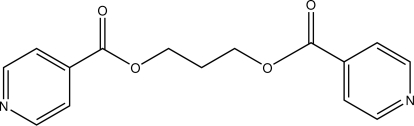


## Experimental

### 

#### Crystal data

C_15_H_14_N_2_O_4_
*M* *_r_* = 286.28
Monoclinic, 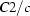

*a* = 23.022 (4) Å
*b* = 4.9336 (5) Å
*c* = 11.9604 (18) Å
β = 98.118 (13)°
*V* = 1344.9 (3) Å^3^
*Z* = 4
Mo *K*α radiation
μ = 0.10 mm^−1^
*T* = 173 K
0.18 × 0.15 × 0.09 mm

#### Data collection

Stoe IPDS II two-circle diffractometer
4231 measured reflections
1251 independent reflections
799 reflections with *I* > 2σ(*I*)
*R*_int_ = 0.070

#### Refinement

*R*[*F* ^2^ > 2σ(*F* ^2^)] = 0.043
*wR*(*F* ^2^) = 0.094
*S* = 0.87
1251 reflections
96 parameters
H-atom parameters constrained
Δρ_max_ = 0.16 e Å^−3^
Δρ_min_ = −0.21 e Å^−3^

### 

Data collection: *X-AREA* (Stoe & Cie, 2001 ▶); cell refinement: *X-AREA*; data reduction: *X-AREA*; program(s) used to solve structure: *SHELXS97* (Sheldrick, 2008 ▶); program(s) used to refine structure: *SHELXL97* (Sheldrick, 2008 ▶); molecular graphics: *XP* (Sheldrick, 2008 ▶); software used to prepare material for publication: *SHELXL97*.

## Supplementary Material

Crystal structure: contains datablocks I, global. DOI: 10.1107/S1600536810011700/fl2297sup1.cif

Structure factors: contains datablocks I. DOI: 10.1107/S1600536810011700/fl2297Isup2.hkl

Additional supplementary materials:  crystallographic information; 3D view; checkCIF report

## Figures and Tables

**Table 1:** Hydrogen-bond geometry (Å, °)

*D*—H⋯*A*	*D*—H	H⋯*A*	*D*⋯*A*	*D*—H⋯*A*
C13—H13⋯N14^i^	0.95	2.65	3.505 (3)	151
C15—H15⋯N14^ii^	0.95	2.72	3.496 (3)	139
C3—H3*A*⋯O1^iii^	0.99	2.98	3.516 (3)	115
C3—H3*B*⋯O1^iv^	0.99	2.62	3.521 (3)	152

Symmetry codes: (i) 
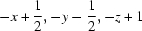
; (ii) 
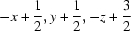
; (iii) 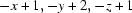; (iv) 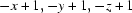.

**Fig. 1.**
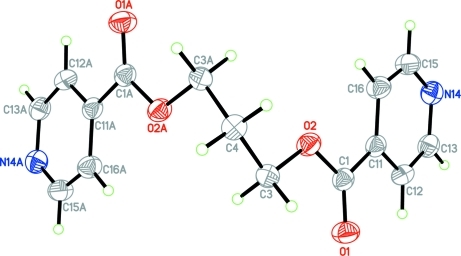
Displacement ellipsoid plot at the 50% probability level for the non-H atoms. Symmetry operator for generating equivalent atoms: (A) 1-x, y, -z+3/2.

**Fig. 2.**
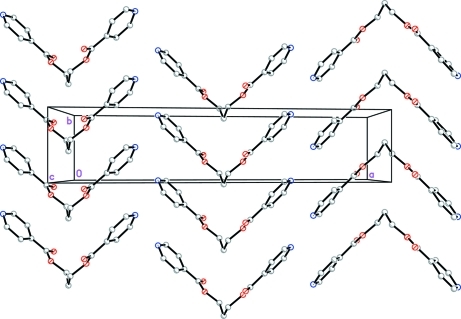
Packing diagram of the title compound with view onto the ab plane.

**Fig. 3.**
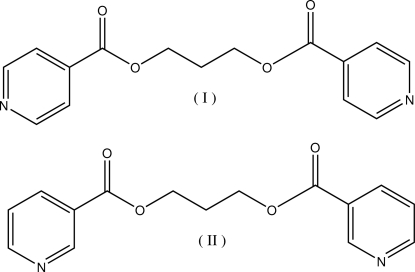
Schematic representations of (I) and (II).

## supplementary crystallographic information

### Comment

The propanediyl group can adopt four possible conformations: trans-trans (TT), trans-gauche (TG), gauche-gauche (GG), and gauche-gauche' (GG') (Carlucci *et al.*, 2002). The title compound C_15_H_14_N_2_O_4, _(I) has a gauche-gauche (GG) conformation and is a positional isomer of the previously reported propane-1,3-diyl bis(pyridine-3-carboxylate), (II), (Brito *et al.*, 2010). Similar compounds have also been reported by Chatterjee *et al.* (2004).

The molecules of the title compound lie on a twofold rotation axis passing through the central carbon atom of the aliphatic chain such that one half of the title compound forms the asymmetric unit (Fig. 1). Both compounds shows excellent agreement of their geometric parameters. The most obvious differences between them are in the torsion angles O/C/C/C—O/C/C/C[56.6 (2)° in (I) and 174.0 (3);70.2 (3)° in (II), GG and TG conformation, respectively] and the angle between the planes of aromatics rings [80.3 (10)° (I) and 76.5 (3)° (II)]. The crystal structure is stabilized by weak C—H··· N and C—H··· O hydrogen bonding (Table 1, Fig. 2). The title compound can be used as a nucleophilic tecton in self-assembly reactions with metal centres of varying lability.

### Experimental

Isonicotinic acid (15 g, 0.122 mol) was stirred in SOCl_2_ (40 ml) in the presence of DMF (0.6 ml) at 60°C for 12 h. Excess thionyl chloride was removed in vacuo. Dried 1,3-Propanediol (4.9 ml, 0.061 mol) was added. After the evolution of hydrogen chloride ended, the mixture was heated at 110°C for 2 h. The mixture was then dissolved in water, and NH_4_OH was added. After filtration, recrystallization in ethyl acetate gave colorless crystals suitable for X-ray analysis. Yield 8.23 g(24%). Analysis calculated for C_15_H_14_N_2_O_4_:*C*: 62.9, H: 4.89, N: 9.68; found: C: 62.45, H: 4.85, N: 9.85. IR (KBr, cm^-1^):(C=O) 1727 s, (C=C) 1596 s, (Ar C—C, C=N) 1408 s, (C—O) 1278 m.

### Refinement

H atoms were placed in idealized positions and treated as riding atoms with C—H distances in the range 0.95-0.99 Å and U_iso_(H) = 1.2U_eq_(C).

### Crystal data

**Table:**

C_15_H_14_N_2_O_4_	*F*(000) = 600
*M**_r_* = 286.28	*D*_x_ = 1.414 Mg m^−^^3^
Monoclinic, *C*2/*c*	Mo *K*α radiation, λ = 0.71073 Å
Hall symbol: -C 2yc	Cell parameters from 2383 reflections
*a* = 23.022 (4) Å	θ = 3.5–25.9°
*b* = 4.9336 (5) Å	µ = 0.10 mm^−^^1^
*c* = 11.9604 (18) Å	*T* = 173 K
β = 98.118 (13)°	Plate, colourless
*V* = 1344.9 (3) Å^3^	0.18 × 0.15 × 0.09 mm
*Z* = 4	

### Data collection

**Table:**

Stoe IPDS II two-circle diffractometer	799 reflections with *I* > 2σ(*I*)
Radiation source: fine-focus sealed tube	*R*_int_ = 0.070
graphite	θ_max_ = 25.6°, θ_min_ = 3.4°
ω scans	*h* = −22→28
4231 measured reflections	*k* = −5→5
1251 independent reflections	*l* = −14→14

### Refinement

**Table:**

Refinement on *F*^2^	Primary atom site location: structure-invariant direct methods
Least-squares matrix: full	Secondary atom site location: difference Fourier map
*R*[*F*^2^ > 2σ(*F*^2^)] = 0.043	Hydrogen site location: inferred from neighbouring sites
*wR*(*F*^2^) = 0.094	H-atom parameters constrained
*S* = 0.87	*w* = 1/[σ^2^(*F*_o_^2^) + (0.0427*P*)^2^] where *P* = (*F*_o_^2^ + 2*F*_c_^2^)/3
1251 reflections	(Δ/σ)_max_ < 0.001
96 parameters	Δρ_max_ = 0.16 e Å^−^^3^
0 restraints	Δρ_min_ = −0.21 e Å^−^^3^

### Special details

**Table:**

Geometry. All esds (except the esd in the dihedral angle between two l.s. planes) are estimated using the full covariance matrix. The cell esds are taken into account individually in the estimation of esds in distances, angles and torsion angles; correlations between esds in cell parameters are only used when they are defined by crystal symmetry. An approximate (isotropic) treatment of cell esds is used for estimating esds involving l.s. planes.
Refinement. Refinement of *F*^2^ against ALL reflections. The weighted *R*-factor wR and goodness of fit *S* are based on *F*^2^, conventional *R*-factors *R* are based on *F*, with *F* set to zero for negative *F*^2^. The threshold expression of *F*^2^ > σ(*F*^2^) is used only for calculating *R*-factors(gt) etc. and is not relevant to the choice of reflections for refinement. *R*-factors based on *F*^2^ are statistically about twice as large as those based on *F*, and *R*- factors based on ALL data will be even larger.

### Fractional atomic coordinates and isotropic or equivalent isotropic displacement parameters (Å^2^)

**Table:**

	*x*	*y*	*z*	*U*_iso_*/*U*_eq_	
O1	0.43077 (7)	0.6064 (3)	0.47123 (12)	0.0356 (4)	
C1	0.41465 (9)	0.5769 (4)	0.56249 (16)	0.0270 (5)	
O2	0.43693 (6)	0.7138 (3)	0.65570 (11)	0.0300 (4)	
C3	0.48573 (9)	0.8943 (4)	0.64276 (16)	0.0287 (5)	
H3A	0.4748	1.0166	0.5775	0.034*	
H3B	0.5204	0.7871	0.6291	0.034*	
C4	0.5000	1.0584 (6)	0.7500	0.0292 (7)	
H4	0.4661	1.1769	0.7579	0.035*	
C11	0.36822 (9)	0.3801 (4)	0.58462 (16)	0.0276 (5)	
C12	0.34752 (9)	0.1948 (4)	0.50109 (17)	0.0299 (5)	
H12	0.3621	0.1949	0.4307	0.036*	
C13	0.30531 (10)	0.0107 (4)	0.52223 (17)	0.0323 (5)	
H13	0.2921	−0.1177	0.4651	0.039*	
N14	0.28183 (8)	0.0018 (4)	0.61815 (14)	0.0337 (5)	
C15	0.30185 (10)	0.1843 (5)	0.69753 (18)	0.0360 (5)	
H15	0.2857	0.1832	0.7663	0.043*	
C16	0.34464 (10)	0.3735 (4)	0.68496 (17)	0.0326 (5)	
H16	0.3577	0.4971	0.7441	0.039*	

### Atomic displacement parameters (Å^2^)

**Table:**

	*U*^11^	*U*^22^	*U*^33^	*U*^12^	*U*^13^	*U*^23^
O1	0.0412 (9)	0.0404 (9)	0.0264 (7)	−0.0035 (8)	0.0090 (6)	−0.0045 (7)
C1	0.0279 (11)	0.0268 (11)	0.0257 (10)	0.0040 (9)	0.0018 (8)	−0.0001 (8)
O2	0.0321 (8)	0.0326 (8)	0.0247 (7)	−0.0054 (7)	0.0021 (6)	−0.0008 (6)
C3	0.0268 (11)	0.0321 (11)	0.0274 (10)	−0.0021 (9)	0.0045 (8)	0.0024 (9)
C4	0.0307 (16)	0.0280 (16)	0.0286 (14)	0.000	0.0034 (12)	0.000
C11	0.0281 (11)	0.0283 (11)	0.0256 (9)	0.0053 (9)	0.0010 (8)	0.0019 (8)
C12	0.0352 (12)	0.0314 (11)	0.0227 (9)	0.0009 (10)	0.0033 (8)	0.0007 (8)
C13	0.0338 (12)	0.0309 (11)	0.0308 (10)	−0.0012 (10)	−0.0001 (9)	−0.0029 (9)
N14	0.0343 (11)	0.0326 (10)	0.0338 (10)	−0.0010 (9)	0.0029 (8)	0.0013 (8)
C15	0.0396 (13)	0.0401 (13)	0.0294 (11)	−0.0008 (11)	0.0085 (9)	−0.0004 (10)
C16	0.0364 (13)	0.0333 (12)	0.0280 (10)	−0.0018 (10)	0.0042 (9)	−0.0050 (9)

### Geometric parameters (Å, °)

**Table:**

O1—C1	1.210 (2)	C11—C12	1.388 (3)
C1—O2	1.342 (2)	C12—C13	1.379 (3)
C1—C11	1.495 (3)	C12—H12	0.9500
O2—C3	1.459 (2)	C13—N14	1.336 (3)
C3—C4	1.513 (2)	C13—H13	0.9500
C3—H3A	0.9900	N14—C15	1.342 (3)
C3—H3B	0.9900	C15—C16	1.381 (3)
C4—C3^i^	1.513 (2)	C15—H15	0.9500
C4—H4	0.9900	C16—H16	0.9500
C11—C16	1.386 (3)		
			
O1—C1—O2	124.0 (2)	C12—C11—C1	118.80 (18)
O1—C1—C11	123.76 (18)	C13—C12—C11	118.75 (19)
O2—C1—C11	112.26 (17)	C13—C12—H12	120.6
C1—O2—C3	115.39 (15)	C11—C12—H12	120.6
O2—C3—C4	108.35 (15)	N14—C13—C12	124.04 (19)
O2—C3—H3A	110.0	N14—C13—H13	118.0
C4—C3—H3A	110.0	C12—C13—H13	118.0
O2—C3—H3B	110.0	C13—N14—C15	116.44 (19)
C4—C3—H3B	110.0	N14—C15—C16	123.7 (2)
H3A—C3—H3B	108.4	N14—C15—H15	118.1
C3^i^—C4—C3	115.3 (2)	C16—C15—H15	118.1
C3^i^—C4—H4	108.3	C15—C16—C11	118.88 (19)
C3—C4—H4	108.5	C15—C16—H16	120.6
C16—C11—C12	118.1 (2)	C11—C16—H16	120.6
C16—C11—C1	123.08 (18)		
			
O1—C1—O2—C3	−3.7 (3)	C16—C11—C12—C13	−1.0 (3)
C11—C1—O2—C3	175.34 (17)	C1—C11—C12—C13	179.05 (19)
C1—O2—C3—C4	172.61 (16)	C11—C12—C13—N14	1.4 (3)
O2—C3—C4—C3^i^	56.56 (11)	C12—C13—N14—C15	−0.7 (3)
O1—C1—C11—C16	−171.1 (2)	C13—N14—C15—C16	−0.5 (3)
O2—C1—C11—C16	9.9 (3)	N14—C15—C16—C11	0.8 (3)
O1—C1—C11—C12	8.9 (3)	C12—C11—C16—C15	0.0 (3)
O2—C1—C11—C12	−170.21 (18)	C1—C11—C16—C15	179.9 (2)

Symmetry codes: (i) −*x*+1, *y*, −*z*+3/2.

### Hydrogen-bond geometry (Å, °)

**Table:**

*D*—H···*A*	*D*—H	H···*A*	*D*···*A*	*D*—H···*A*
C13—H13···N14^ii^	0.95	2.65	3.505 (3)	151
C15—H15···N14^iii^	0.95	2.72	3.496 (3)	139
C3—H3A···O1^iv^	0.99	2.98	3.516 (3)	115
C3—H3B···O1^v^	0.99	2.62	3.521 (3)	152

Symmetry codes: (ii) −*x*+1/2, −*y*−1/2, −*z*+1; (iii) −*x*+1/2, *y*+1/2, −*z*+3/2; (iv) −*x*+1, −*y*+2, −*z*+1; (v) −*x*+1, −*y*+1, −*z*+1.
